# Evaluating the treatment of metastatic colorectal cancer with monoclonal antibodies

**Published:** 2012-06-18

**Authors:** C Popa, S Ionescu, D Mihăilă, I Gal, T Potecă, S Simion

**Affiliations:** * General Surgery Clinic, “Colentina” Clinic Hospital, Bucharest, Romania, “Carol Davila” University of Medicine and Pharmacy, Bucharest, Romania; ** General and Oncologic Surgical Clinic, Bucharest Oncologic Institute, Bucharest, Romania; *** General Surgery Clinic, “Colentina” Clinic Hospital, Bucharest, Romania

**Keywords:** biologic therapy, tumor biomarkers, monoclonal antibodies, K-RAS mutations, metastatic colorectal cancer

## Abstract

The ability to tailor biologic therapy based on the status of tumor biomarkers and monoclonal antibodies has become very important in the last years. The role of tumor biomarkers in treating colorectal cancer, specifically the K-RAS gene, was identified. K-RAS had a higher interest after Lievre and colleagues reported at the 2008 American Society of Clinical Oncology (ASCO) meeting, their analysis of K-RAS mutations in tumors from patients who did not appear to benefit from cetuximab therapy, providing additional data involving K-RAS mutant tumors and their lack of response to cetuximab, as part of first-line therapy for metastatic colorectal cancer. Furthermore, other trials evaluated the K-RAS status and the first-line treatment of metastatic colorectal cancer, the treatment of refractory metastatic cancer and dual-antibody therapy in the first-line treatment of colorectal cancer. Patients with mutant K-RAS colorectal tumors have no benefit from cetuximab, no matter the type of chemotherapy regimen.

## Background

In 2004, bevacizumab became the first monoclonal antibody targeted to vascular endothelial growth factor (VEGFR). Subsequently, new types of monoclonal antibodies, targeted to the epidermal growth factor receptor (EGFR), as cetuximab and panitumumab, have been discovered. 

Bevacizumab is a recombinant humanized monoclonal antibody directed against the vascular endothelial growth factor (VEGF), a pro-angiogenic cytokine. 

Cetuximab is a chimeric monoclonal antibody indicated for use in patients who have progressed on irinotecan-based therapy or are intolerant to irinotecan-based therapy. 

Panitumumab is a fully human monoclonal antibody indicated for use as a single agent in patients who have progressed on or following fluoropyrimidine-, oxaliplatin-, and irinotecan-containing chemotherapeutic regimens.

### The Evolving Role for K-RAS in the Choice of Biologic Agents for Metastatic Colorectal Cancer

Bevacizumab binds to VEGF and inhibits VEGF receptor binding (a pro-angiogenic cytokine), thereby preventing the growth and maintenance of tumor blood vessels. 

In patients with colorectal cancer, responses to EGFR-targeted therapy appear to be independent of the level of EGFR expression. Thus, other molecular mechanisms may influence the efficacy of response to these agents. 

Cetuximab and panitumumab are monoclonal antibodies that bind to the epidermal growth factor receptor (EGFR) and inhibit ligand-induced phosphorylation of EGFR. Cetuximab in combination with irinotecan-based chemotherapy improve response rates (RR) and time to progression in patients who are refractory to the treatment with irinotecan. K-RAS is a protein in the downstream intracellular signaling pathway of the EGFR involved in cell differentiation, proliferation, and angiogenesis. Mutations in the K-RAS gene cause the activation of K-RAS even in the absence of EGFR ligand binding, thereby rendering antibodies targeting the upstream EGFR is ineffective [**1**].

The RAS protein is a molecule involved in EGFR signaling; it plays a central role in other intracellular signaling pathways. In normal cells, RAS acts as a molecular on/off switch. RAS proteins cycle between a guanosine diphosphate (GDP)-bound off state and a guanosine triphosphate (GTP)-bound on state. By acting as molecular switches, these proteins link extracellular signals initiated by cell surface receptors, such as EGFR, and transmit them downstream to the nucleus of the cell [**2**].

**Fig. 1:**
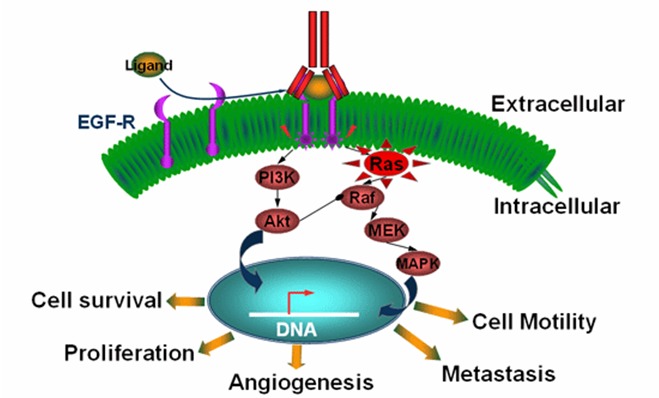
The Role of RAS Protein in EGFR Signaling Pathway (Source: Medscape General Surgery)

When the EGF ligand binds to the EGFR, EGFR dimerizes, becomes activated and then transmits a phosphorylation signal to RAS. Once activated by phosphorylation, RAS in turn phosphorylates other downstream proteins, and through a cascade of sequential phosphorylation events, gene expression is altered. As a result of EGFR activation, these changes in gene expression can lead to cell proliferation, resistance to apoptosis, angiogenesis, cell motility, and metastasis (**Fig. 1**).

In the absence of growth factors, a single amino acid change in the RAS proteins can make them be permanently switched on. These mutations most commonly occur in codons 12, 13, and 61, and they either destroy GTPase activity (the enzyme that removes a phosphate molecule and turns RAS from “on” to “off”) or prevent GTPase-associated protein binding, which induces the hydrolysis of GTP (on) to GDP (off). The mutated RAS then becomes locked in an “on state” and is therefore activated independently of EGFR signaling.

EGFR-targeted agents block the activation of the EGFR receptor at the initial step in the cascade and have the potential to stop RAS signaling. However, a mutated RAS gene, producing a RAS molecule that is permanently switched on, is likely to be unaffected by EGFR-targeting agents. Clinical data support the following idea: patients whose tumors have wild-type RAS show a far greater response to EGFR inhibitors compared with those with mutated RAS, which is permanently switched on [**2**].

### The Role of RAS in Colorectal Cancer Progression

The RAS proteins, most notably K-RAS, play an important role in colorectal cancer progression. Fearon and Vogelstein defined a multistep genetic model for the formation of colorectal cancer. According to this model, K-RAS mutations, along with other mutations, are necessary, although not sufficient, for a cancer to progress from a small to a large adenoma. The colorectal cancer develops after a series of molecular alterations have accumulated, including K-RAS, p53, and adenomatous polyposis coli. Not all mutations are necessary for neoplasia to develop [2].

Amado and colleagues published the first demonstration of K-RAS mutations as a negative predictive marker for EGFR antibodies in a randomized trial. While using panitumumab vs. the best supportive care as last-line therapy, the researchers observed a statistically significant improvement in progression-free survival (PFS) in the panitumumab arm overall compared with the best supportive care (8.0 weeks and 7.3 weeks). This effect was more pronounced among patients with K-RAS wild-type (normal) status (median PFS of 12.3 weeks in the panitumumab arm vs. 7.3 weeks for best supportive care). No benefit was observed from adding panitumumab among patients with K-RAS mutant status. K-RAS suffered mutations in 40% of the colorectal cancer cases [**1,2**].

Several hypotheses have arisen: 

a). K-RAS mutations affect the efficacy of cetuximab, including responses to combination chemotherapy and cetuximab therapy in the first-line setting 

b). is cetuximab beneficial, in combination with standard chemotherapy, in patients with the K-RAS mutation? 

In 2006, Lièvre and colleagues have studied the tumors from 30 patients treated with cetuximab, who were screened for K-RAS, B-Raf, and phosphatidyl-inositol 3 kinase (PI3K) mutations as well as EGFR status. From all the patients, 37% responded to cetuximab. None of the 11 responding patients had K-RAS mutations compared with 68.4% of the 19 nonresponding patients. In addition, survival was also significantly extended in patients with wild-type K-RAS compared with mutated K-RAS. An increased EGFR copy number, present in only 3 patients, was also significantly associated with a response to cetuximab. This study was reloaded by Lievre on 89 de cases with metastatic colorectal cancer, after they had failed irinotecan-based therapy, obtaining the same results [**2**].

In 2008, Di Fiore and colleagues reported the findings of a meta-analysis that included 281 patients from 7 studies. Patients who had irinotecan-refractory metastatic colorectal cancer were treated with cetuximab plus irinotecan-based chemotherapy. 77 (27.4%) responded to therapy (3 complete responses), stable disease (SD) was observed in 107 (38.1%), and progressive disease (PD) in 97 (34.5%). No responses were observed among the patients with tumors with K-RAS mutations, and 59.8% had progressive disease (PD) [**1,2**].

### K-RAS Status and First-Line Treatment of Metastatic Colorectal Cancer

The CRYSTAL trial compared the use of folinic acid/fluorouracil/irinotecan (FOLFIRI) vs. FOLFIRI plus cetuximab in the first-line treatment of metastatic colorectal cancer. The initial results of the CRYSTAL trial, presented by Van Custem in 2007, showed that the addition of cetuximab to FOLFIRI increased RR by approximately 8% and prolonged PFS to 0.9 months (without selection for K-RAS status). In response to the emerging data showing a correlation between mutations in K-RAS and lack of clinical benefit with cetuximab, a retrospective analysis was performed to evaluate the impact of K-RAS mutations on PFS and RR using data from CRYSTAL study. Quantitative PCR analysis for the K-RAS mutation was performed on archived tumor tissue from patients in the CRYSTAL trial, and was available for evaluation in 45%. There were 64.4% subjects with K-RAS wild-type tumors and 35.6% with K-RAS mutant tumors. In the K-RAS wild-type population, median PFS for cetuximab plus FOLFIRI was of 9.9 months vs. 8.7 months for FOLFIRI alone (P = 0.017). There was also a 16% increase in overall RR in the K-RAS wild-type group that received cetuximab plus FOLFIRI. In contrast, there was no benefit at all in RR or PFS among patients with mutant K-RAS receiving FOLFIRI plus cetuximab vs. FOLFIRI alone.

This study provides clear evidence that patients with K-RAS wild-type tumors have a benefit from the addition of cetuximab to FOLFIRI than those with K-RAS mutant tumors. Moreover, patients with K-RAS mutant tumors had no improvement in RR or PFS when cetuximab was added to FOLFIRI; it can be concluded that cetuximab should not be used to these individuals for first-line treatment of metastatic colorectal cancer [**1-5**].

The OPUS trial evaluated the use of 5-fluorouracil/leucovorin/oxaliplatin (FOLFOX)4 vs. FOLFOX4 plus cetuximab in the first-line treatment of metastatic colorectal cancer; the primary endpoint was RR. According to the initial results, the addition of cetuximab did not significantly improve PFS or RR compared with FOLFOX alone. When stratified for performance status (PS), the RR was significantly improved with the addition of cetuximab. In the K-RAS wild-type group, the addition of cetuximab to FOLFOX4 significantly increased RR (61%) compared with FOLFOX4 alone (37%). However, in the K-RAS mutant group, RR showed a trend toward a worse rate in the cetuximab plus FOLFOX4 arm (33%) vs. FOLFOX4 alone (49%). Among patients with K-RAS wild-type tumors, median PFS was significantly improved with FOLFOX plus cetuximab vs. FOLFOX4 alone (7.7 months vs. 7.2 months). In contrast, median PFS significantly worsened in patients who received FOLFOX4 plus cetuximab vs. FOLFOX4 alone (5.5 months vs. 8.6 months) in the K-RAS mutant group. 

The OPUS investigators concluded that RR and PFS were significantly improved with the addition of cetuximab to FOLFOX4 in patients with K-RAS wild-type tumors. With the use of cetuximab plus FOLFIRI, patients with mutated K-RAS appeared to derive no benefit from the combination of cetuximab and FOLFOX [**1,2,6-8**].

The BEAT Study (Bevacizumab Expanded Access Trial) evaluates the safety and efficacy of bevacizumab plus routine first-line chemotherapy regimens in a large patient population with metastatic unresectable colorectal cancer. Patients received chemotherapy plus bevacizumab [5 mg/kg every 2 weeks (5-fluorouracil regimens) or 7.5 mg/kg every 3 weeks (capecitabine regimens)]. The primary endpoint was safety. Secondary objectives were progression-free survival (PFS) and overall survival (OS).

The final analysis comprised 1914 assessable patients (males 58%; median age 59 years). Chemotherapy included 5-fluorouracil/leucovorin (5-FU/LV) + oxaliplatin (29%), irinotecan plus 5-FU/LV (26%), capecitabine plus oxaliplatin (18%) and monotherapy (16%). Serious/grade 3–5 adverse events of interest for bevacizumab included bleeding (3%), gastrointestinal perforation (2%), arterial thromboembolism (1%), hypertension (5.3%), proteinuria (1%) and wound-healing complications (1%). Sixty-day mortality was of 3%. Median PFS was of 10.8 months [monotherapy - 8,6 months, FOLFIRI 11,6 months, FOLFOX 11,3 months, XELOX 10,8 months] and median OS reached 22.7 months (FOLFIRI 23,7 months, FOLFOX 25,9 months, XELOX 23 months; monotherapy - 18 months).

The BEAT study shows that the efficacy and safety profile of bevacizumab in routine clinical practice is consistent with results observed in another large observational study (BriTE) [**9**].

### K-RAS Status and the Treatment of Refractory Metastatic Colorectal Cancer

The EVEREST trial was designed to determine whether an escalation of cetuximab in combination with a standard dose of irinotecan could improve the efficacy in patients who failed irinotecan-based therapy. After 22 days of treatment, patients with grade 0/1 skin reactions were randomized to continue to receive irinotecan (180 mg/m2 biweekly) plus standard dose cetuximab (250 mg/m2 weekly) in arm A, or irinotecan (180 mg/m2 biweekly) plus escalated doses of cetuximab (50 mg/m2 every 2 weeks up to 500 mg/m2 weekly) in arm B. Data from the trial, presented by Tejpar and colleagues, in 2007, showed that the dose escalation of cetuximab, improved the response rate (RR). It was also associated with a doubling of grade 3/4 diarrhea and grade 2 or higher skin rash.

For patients with wild-type K-RAS, 21.1% who received standard cetuximab had a response vs. 46.4% who received escalated cetuximab doses. However, none of those with K-RAS mutations in either arm achieved a response with the combination of cetuximab plus irinotecan [**1,2,10**].

The results from this analysis confirm the findings of previous retrospective studies (OPUS, CRYSTAL, and PACCE): patients with K-RAS mutant colorectal tumors do not benefit from the addition of cetuximab. Colorectal tumors should be evaluated for the presence of the K-RAS mutation. If the mutation is present, the patient should not be offered cetuximab. K-RAS mutation testing will spare those who will not benefit from additional toxicity and expense of cetuximab, while also ensuring that those with K-RAS wild-type tumors, who may benefit, can receive cetuximab or panitumumab. Indeed, the European regulatory agency (EMEA) has restricted the approval of cetuximab and panitumumab in colorectal cancer to patients with wild-type K-RAS tumors [**1,3-8,11**].

### Dual-Antibody Therapy in the First-Line Treatment of Colorectal Cancer

Based on preclinical models, it is suggested that the inhibition of the vascular endothelial growth factor (VEGF) combined with bevacizumab, and EGFR with cetuximab, has additive effects. Data from the BOND2 study demonstrated that the use of the 2 agents in combination with irinotecan-based chemotherapy is feasible and potentially more efficacious than irinotecan plus cetuximab alone among patients refractory to irinotecan-based therapy. Results of the PACCE trial, which compared FOLFOX or FOLFIRI plus bevacizumab with or without panitumumab, however, showed inferior PFS with panitumumab. These surprising results were initially attributed to increased toxicity in the panitumumab arms [**11,12**].

The CAIRO2 study used a similar approach as PACCE, investigating the effect of adding cetuximab to capecitabine, oxaliplatin, and bevacizumab in patients with advanced colorectal cancer. Earlier in 2008, Tol and colleagues reported the toxicity analysis of CAIRO2, demonstrating that the dual biologic therapy did not lead to excessive or unexpected toxicity. Efficacy results were presented by Punt and colleagues at ASCO 2008, at a median follow-up period of 18.7 months [**13**].

Patients were randomized to 1 of 2 arms. Arm A received oxaliplatin 130 mg/m² day 1, capecitabine 1000 mg/m² twice a day, days 1-14, bevacizumab 7.5 mg/kg day 1, repeated every 3 weeks. Oxaliplatin was discontinued after cycle 6. Arm B received oxaliplatin, capecitabine, and bevacizumab as in arm A, with the addition of cetuximab weekly 250 mg/m² (400 mg/m² first dose). Evaluation of tumor response was repeated every 3 cycles. Median PFS was significantly reduced in arm B (addition of cetuximab) from 10,7 months to 9.6 months. No differences in RR or OS were seen between the groups. All adverse effects were significantly worsened in arm B, but toxicity, as a reason for discontinuation of treatment, did not significantly differ between treatment arms. The median number of cycles administered was also reduced in the cetuximab-containing arm.

The investigators also evaluated the effect of K-RAS mutation on PFS and overall survival (OS). Among patients with K-RAS mutant tumors, the addition of cetuximab significantly shortened median PFS (8.6 months vs. 12.5 months); OS was not different. There was no difference in PFS or OS between arm A and B in those with K-RAS wild-type tumors. Nor was there a statistically significant decrease in median PFS or OS between those with K-RAS wild type and K-RAS mutant tumors in the cetuximab arm.

The CAIRO2 authors concluded that the addition of cetuximab to irinotecan, capecitabine, and bevacizumab significantly reduced PFS, but this did not have an impact on OS. Toxicities were also increased in the cetuximab arm, but were considered acceptable. Among patients with K-RAS mutant tumors, the addition of cetuximab was associated with a decrease in PFS, but again, has not significantly affected OS [**1,2,13**]. 

Although the initial reports of dual antibody therapy (cetuximab/bevacizumab) in a last-line setting were intriguing, the findings of CAIRO2 confirm and validate the data from PACCE, which indicated that the addition of EGFR antibodies to a bevacizumab-containing first-line regimen does not increase efficacy and is likely associated with inferior outcome. In first-line therapy, dual antibody therapy should not be used for instance [**1**]. 

## Conclusions

1. As a result of EGFR activation, these changes in gene expression can lead to cell proliferation, resistance to apoptosis, angiogenesis, cell motility, and metastasis.

2. EGFR-targeted agents block the activation of the EGFR receptor and stop Ras signaling; a mutated Ras gene, producing a Ras molecule that is permanently switched on, is unaffected by EGFR-targeting agents.

3. Patients with mutant K-RAS colorectal tumors have no benefit from the addition of cetuximab, no matter the type of chemotherapy regimen: first line or refractory metastatic colorectal cancer.

4. EMEA has restricted approval of cetuximab and panitumumab in colorectal cancer to patients with wild-type K-RAS tumors.

5. The use of EGFR antibodies to a bevacizumab-containing a first-line regimen does not increase the efficacy and is likely associated with an inferior outcome.
